# The implication of leaf anatomical structure for the selective breeding of lilacs

**DOI:** 10.18699/VJ21.060

**Published:** 2021-09

**Authors:** L.M. Pshennikova

**Affiliations:** Botanical Garden-Institute of the Far Eastern Branch of the Russian Academy of Sciences, Vladivostok, Russia

**Keywords:** Syringa oblata, Syringa vulgaris, Pseudocercospora lilacis, leaf anatomical structure, adaptation, interspecif ichybridization, Syringa oblatа, Syringa vulgaris, Pseudocercospora lilacis, анатомическое строение листа, адаптация, межвидовая гибридизация

## Abstract

The cultivars of the common lilac (Syringa vulgaris) grown in the south of the Russian Far East are not always winter-hardy and are often damaged by fungal diseases due to a very humid climate. A promising trend in the selective breeding of lilacs in Russia is the creation of new breeding material based on the gene pool of the broadleaf lilac (S. oblata) and its hybrids in order to introduce valuable adaptive traits into cultivars. The present work aimed to identify the traits of leaf anatomy in species and cultivars of Syringa resistant and susceptible to Pseudocercospora lilacis, the causative agent of brown leaf spot disease. The study was carried out on the living collection of the Botanical Garden-Institute, Far Eastern Branch, Russian Academy of Sciences (Vladivostok). The leaf anatomical structure of two Syringa species showing different degrees of resistance to P. lilacis in the monsoon climate of the Far East (resistant S. oblata and weakly resistant S. vulgaris, and also their hybrid cultivars) has been analyzed. The differences between species, subspecies, and cultivars are quantitative: they differ in the number of spongy mesophyll layers, the cell height in the f irst layer of palisade mesophyll, the cell height in the upper and lower epidermises, and the thickness of both mesophylls. The interspecif ic hybrids resistant or weakly resistant to P. lilacis (brown leaf spot disease) mainly retain the leaf anatomy structure of the maternal plant. One of the traits determining the resistance of hybrid lilac cultivars is an increased number of spongy mesophyll layers in the leaf blade. The study of leaf anatomy has shown that the four-layered spongy mesophyll leaf parenchyma correlates with the resistance of lilacs from the subsection Euvulgaris to P. lilacis. In S. oblata, this trait is inherited down the maternal line. To establish lilac cultivars resistant to fungal diseases, it is advisable to cross the two species (S. oblata and S. vulgaris) or their cultivars using one of S. oblata subspecies as a maternal plant.

## Abstract (ru)

Сорта сирени обыкновенной (Syringa vulgaris) на юге российского Дальнего Востока не всегда зимостой- ки, повреждаются грибными болезнями, что связано с избыточной влажностью климата. Перспективным направ- лением отечественной селекции сиреней является создание нового селекционного материала с использованием генофонда Syringa oblata и ее гибридов для введения в культурные сорта ценных адаптивных признаков. Целью ра- боты было выявление анатомических особенностей листьев видов и сортов рода Syringa, устойчивых и восприимчи- вых к возбудителю бурой листовой пятнистости Pseudocercospora lilacis. Исследования проведены на коллекционном материале открытого грунта Ботанического сада-института ДВО РАН. Проанализировано анатомическое строение листа двух видов Syringa, различающихся по устойчивости к P. lilacis в муссонном климате Дальнего Востока: устой- чивого S. oblata и слабоустойчивого S. vulgaris, а также гибридных cортов, полученных на их основе. Различия между видами, подвидами и сортами носят количественный характер: число рядов губчатой паренхимы, высота клеток первого ряда палисадной паренхимы, высота клеток верхней и нижней эпидермы, толщина палисадного и губчато- го слоя. Устойчивые и малоустойчивые к P. lilacis межвидовые гибриды преимущественно сохраняют анатомическое строение листа материнского растения. Одним из определяющих признаков устойчивости гибридных сортов сире- ней является повышенное количество рядов губчатой паренхимы листовой пластинки – признак, передающийся от S. oblata по материнской линии. Создание устойчивых к грибным заболеваниям сортов сиреней целесообразно проводить путем скрещивания видов S. oblata и S. vulgaris или их сортов, используя в качестве материнского рас- тения один из подвидов S. oblata. В ходе исследования анатомии листа установлено, что губчатая ткань из четырех рядов коррелирует с устойчивостью сиреней из подсекции Euvulgaris Schneid. к P. lilacis на юге Приморского края. Этот признак листа S. oblata передается по наследству по материнской линии.

## Introduction

The use of new cultivars that are resistant to pathogenic biota is a solution to problems of not only economic, but also environmental significance. Lilac species and cultivars have long been recognized as valuable ornamental plants. However, the habitat and climatic conditions of Vladivostok and its suburbs are very specific (Agroclimatic Resources…, 1973), which becomes a serious obstacle to the introduction of many exotic, alien trees and shrubs in southern Primorsky Krai, Russia, and their outdoor cultivation. The present study was conducted to extend our knowledge about the mechanisms of plant adaptation to the specific climate in the south of the Russian Far East such as, in particular, the mechanisms of protection against adverse biotic factors of the environment shown by some cultivars from the subsection Euvulgaris Schneid. of the genus Syringa L. There are a number of works published by various botanical institutions that elucidate the species composition of the pathogenic biota associated with the genus Syringa (Khomyakov, Tereschenko, 2000; Tomoshevich, Vorobjova, 2010; Chervyakova, Keldish, 2018; Pavlenkova, 2018; Polyakova, 2018, etc.). We could not find any studies that consider the factors of lilacs’ resistance or susceptibility to fungal diseases, and there is also a lack of recent data on the mechanism of lilacs’ resistance to fungal diseases.

According to our observations (Pshennikova, 2007, 2018), the most resistant cultivars in southern Primorsky Krai are those from the garden group Hyacinthiflora, which have been obtained through interspecific hybridization of S. oblata and S. vulgaris, freely interbreeding with each other. However, this group also includes cultivars that differ in the degree of susceptibility to pathogenic fungi.

The broadleaf lilac, Syringa oblata Lindl., is an introduced plant in Primorsky Krai, brought by S.I. Elovitsky from China in the early 20th century (Vasilyuk et al., 1987). In nature, it is found in the northern part of Northeast China (Saakov, 1960; Mei-chen et al., 1996). Currently it is often used for decorating the landscape of the city of Vladivostok and other populated areas of Primorsky Krai. This species is resistant not only to the winter conditions of the region, but also to pests and fungal diseases, and, apparently, has immunity acquired during the evolution in similar climatic conditions of China. Some interspecific hybrid cultivars possess this resistance (Pshennikova, 2007, 2018).

The common lilac, Syringa vulgaris L., a species close to S. oblata, is winter-hardy in the conditions of Primorsky Krai. It was introduced into Primorsky Krai in the mid-20th century, probably, from Chernigov Oblast, Ukraine (Vasilyuk et al., 1987). Under the continental climate of its natural habitats in highlands (cretaceous slopes on the Balkan Peninsula), this plant has developed a high drought resistance and tolerance to sudden temperature variations. However, for the same reason, it has not formed mechanisms of protection from high humidity characteristic of the climate in southern Primorsky Krai, which is one of the factors responsible for the high prevalence of pathogenic fungi damaging the species and its cultivars. The lilac brown leaf spot disease (Bunkina et al., 1971) causes especially serious damage to this lilac, resulting in loss of decorative appearance of bushes and premature leaf fall.

The features of leaf anatomical structure in pathogen-resistant species and cultivars are considered to be the primary barriers or passive immunity factors (Vavilov, 1964; Shkalikov et al., 2005; Plotnikova, 2007; Shestakova, 2010, 2013). Leaf traits (pubescence, the thick cuticular layer, the thick epidermis, and also the anatomical specifics of mesophyll) have a significant effect on plant immunity (Furst, 1968; Pautov et al., 2002; Sokolova, 2010; Motyleva, Dzhigadlo, 2012). The first data on specifics of the leaf apparatus structure in hybrid cultivars of ornamental woody plants in the literature date back to the 1970s–1980s (Eremin, Novikova, 1976; Novikova, 1976, 1982; Pham van Nang, 1976; Turovsky et al., 1978; Bykova, 1979).

The present study aimed to identify the features of leaf blade anatomical structure in the Syringa species and cultivars, bred on the basis of S. oblata and S. vulgaris, which differ in the degree of resistance to Pseudocercospora lilacis (Desm.) Deighton

## Materials and methods

A total of 22 representatives of subsection Euvulgaris Schneid. of the genus Syringa L. (Table 1) from the live collection grown on open-air plots of the Botanical Garden-Institute, Far Eastern Branch, Russian Academy of Sciences (BGI FEB RAS), located in the coastal zone of southern Primorsky Krai, were used as objects of the study. The material was collected from 2016 to 2019.

**Table 1.**
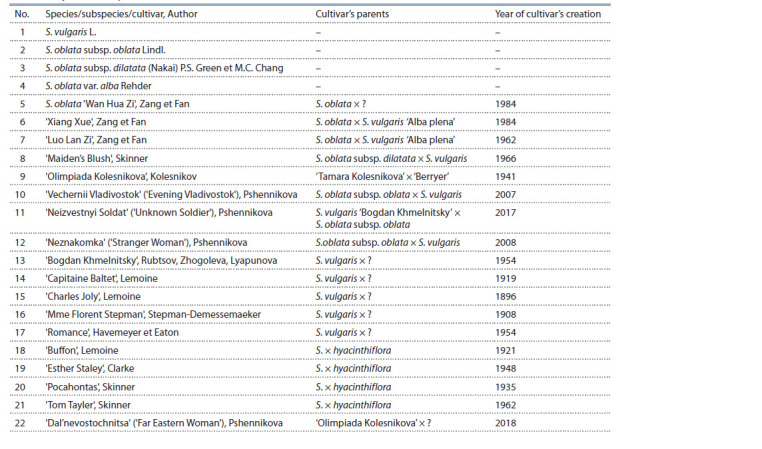
Objects of study

The degree of resistance of lilac species and cultivars to P. lilacis was scored on a 5-point scale for ornamental cultivated plants (Tamberg, Ulyanova, 1969), which we adapted for the genus Syringa (see Table 2): (1) no disease or up to 10 % of leaf surface of the plant damaged; (2) up to 25 % of leaf surface damaged; (3) up to 50 % of leaf surface damaged; (4) up to 75 % of leaf surface damaged; (5) over 75 % of leaf surface damaged.

To analyze mesophyll, five leaf blades were used. The third leaf from the base of a vegetative shoot, completely grown, was sampled from the southern aspect of crown. Leaves were fixed in 70 % ethyl alcohol. Cross-sections through the middle part of a leaf blade between the midrib and the leaf edge were cut on a freezing microtome, stained with a safranin solution, and embedded in glycerol/jelly. The sections were examined under a Zeiss Axioplan 2 Imaging microscope (Carl Zeiss, Germany) using the AxioVision 4 software. The data obtained were processed in the MS Excel package

**Fig. 1.**
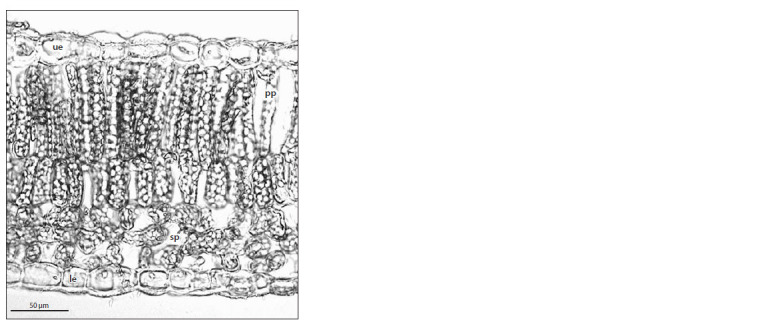
A leaf cross-section from the lilac cultivar ‘Neizvestnyi Soldat’. The letter designations are as follows: ue, upper epidermis; pp, palisade mesophyll parenchyma; sp, spongy mesophyll parenchyma; le, lower epidermis.

The following anatomical characters of a leaf were considered: leaf thickness, height of upper and lower epidermis, thickness of palisade mesophyll, thickness of spongy mesophyll, number of layers of spongy and palisade mesophylls, and size of cells in the 1st and 2nd layers of palisade mesophyll in hybrid cultivars (Fig. 1). The study was conducted at the Center for Collective Use “Microtechnical Laboratory”, BGI FEB RAS, and “Bioresourse Collection”.

Statistical analysis was carried out using the STATISTICA 6.0 software package. The data were tested for normality of distribution using the Shapiro–Wilk W-test (Shapiro, Wilk, 1965). To search for a statistical relationship between the variables, a correlation analysis was performed using the Spearman Rank Order Correlations (Fieller et al., 1957). Linear measurements of leaf blade tissues were assumed to be independent variables. Degree of resistance of a species/ cultivar to P. lilacis was assumed to be a dependent variable. According to the W-test, the distribution of analyzed data differed from the normal one ( p-value ˂ 0.05). Measurements for each representative were made in 20 to 30 replicates; the total number of observations was 454.

## Results and discussion

Long-term observations on the species and cultivars of the genus Syringa made it possible to arrange them in the order of increasing degree of their leaves’ resistance to P. lilacis (Table 2).

**Table 2.**
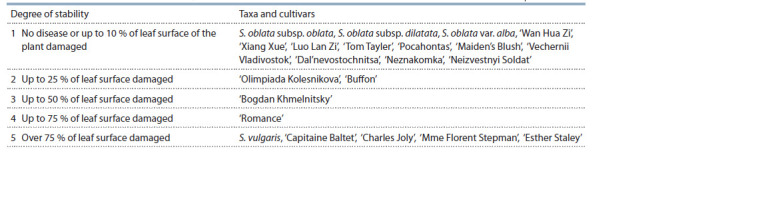
The degree of resistance of the Syringa varieties and species to the fungal diseases Pseudocercospora lilacis

**Fig. 2.**
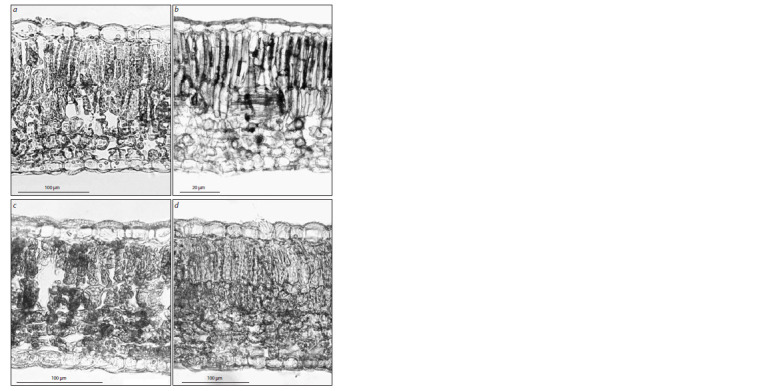
Leaf cross-sections from the following lilacs: a, S. vulgaris; b, S. oblata subsp. oblata; c, S. oblata subsp. dilatata; d, S. oblata var. alba.

The first group included two subspecies of S. oblatа and their hybrid cultivars. Subspecies of S. oblatа differ in the shape and size of leaf blade. The study of the leaf anatomy of S. oblatа and S. vulgaris allowed identification of the distinguishing traits of these species (Fig. 2, Table 3).

**Table 3.**
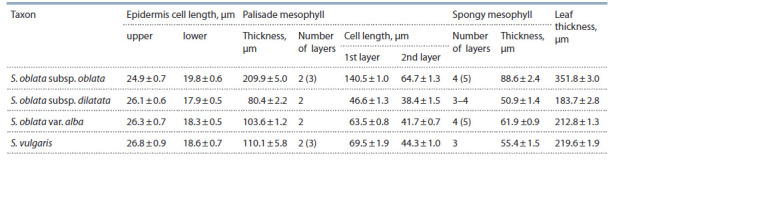
Characteristics of leaf cross-sections from species of the genus Syringa

According to the data obtained, the species S. oblata resistant to P. lilacis differs from the non-resistant species S. vulgaris by an increased number of spongy mesophyll layers. The subspecies of S. oblata differ from one another in the thickness of leaf blade and the height of palisade mesophyll cells. S. oblata subsp. oblata has cells of the upper layer of palisade mesophyll almost twice as large as the second layer cells. The revealed difference may be a systematic trait of S. oblata subsp. oblata on the anatomical level. In S. oblata subsp. dilatata, the heights of the layers of palisade mesophyll cells are either equivalent, or the first layer is slightly, almost 1.2-fold, larger than the second one (see Fig. 2, c). This subspecies has upper epidermis cells as large as those in S. vulgaris.

We found that the thickness of leaf epidermis and mesophyll in the species under study are not related to resistance against P. lilacis (see Table 3). Thus, the height of the upper epidermis cells in the weakly resistant species S. vulgaris is greater than that in the fungus-immune subspecies S. oblata subsp. oblata, or the ratio of the 1st and 2nd layers of palisade mesophyll in S. vulgaris is the same as in the subspecies S. oblata subsp. dilatata resistant to the fungal pathogen. The number of palisade mesophyll layers is not a constant trait in these species and can vary from 2 to 3. The subspecies S. oblata subsp. dilatata has the leaf anatomical traits close to those of S. vulgaris and S. oblata subsp. oblata.

**Fig. 3.**
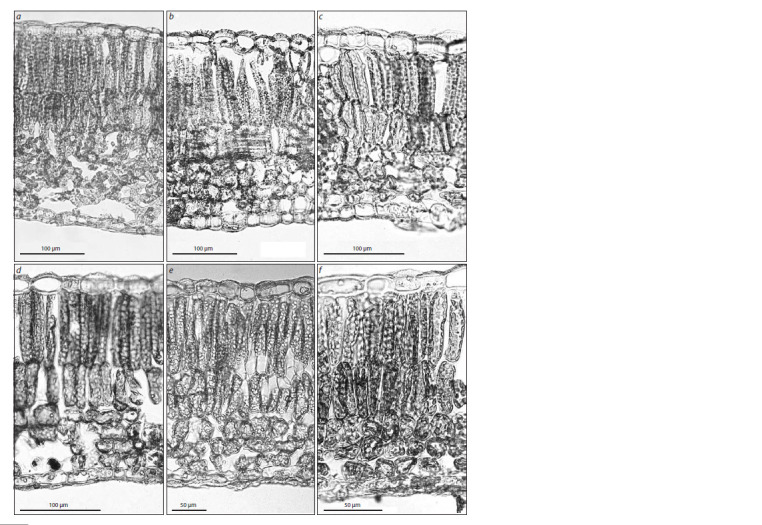
Leaf cross-sections from lilacs of the following cultivars: a, ‘Vechernii Vladivostok’; b, ‘Wan Hua Zi’; c, ‘Maiden’s Blush’; d, ‘Neznakomka’; e, ‘Olimpiada Kolesnikova’; f, ‘Bogdan Khmelnitsky’.

The following group of lilacs combines cultivars obtained through hybridization of two species, S. oblata (maternal species) and S. vulgaris. The cultivars ‘Vechernii Vladivostok’, ‘Neznakomka’, ‘Luo Lan Zi’, and ‘Wan Hua Zi’ repeat the structural features of S. oblata subsp. oblata; they do not differ in the number of layers of palisade and spongy mesophylls (Table 4, Fig. 3, a‒d ); these cultivars are resistant to P. lilacis. It has been found that the S. oblata cultivars are distinguished by the height of the upper and lower epidermises, the thicknessof palisade and spongy mesophyll parenchyma, and the leaf thickness. The resistant cultivars are characterized by the increased number of spongy mesophyll layers, 4 or more. 

**Table 4.**
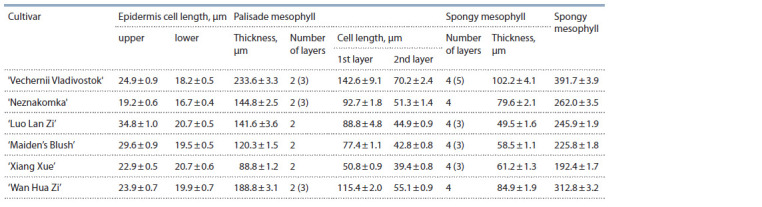
Characteristics of leaf cross-sections from the S. oblata cultivars

The cultivars ‘Xiang Xue’ and ‘Maiden’s Blush’ are also resistant to P. lilacis. Their leaf anatomical structure is characteristic of S. oblata subsp. dilatata. Their spongy mesophyll is mostly 4-layered, but 3 layers are also observed sometimes.

Another group under study combined the cultivars with both parents being S. vulgaris: ‘Capitaine Baltet’, ‘Charles Joly’, ‘Mme Florent Stepman’, ‘Romance’, and ‘Bogdan Khmelnitsky’, which proved to be non-resistant to P. lilacis to varying degrees. Their leaves are medium in thickness, the palisade mesophyll is 2-layered, and the spongy mesophyll is 3-layered. In ‘Bogdan Khmelnitsky’, the spongy mesophyll consists sometimes of 4 layers of cells (see Fig. 3, f ). This can apparently be explained by the fact that the species S. oblata was used for breeding the parental cultivars of ‘Bogdan Khmelnitsky’ at some stage in the past. Compared to the other S. vulgaris cultivars, this one is more resistant to the fungal pathogen. The cultivars differ in the height of cells of upper and lower epidermises and in the thickness of palisade and spongy mesophylls. The leaf structure is typical of S. vulgaris (Table 5).

**Table 5.**
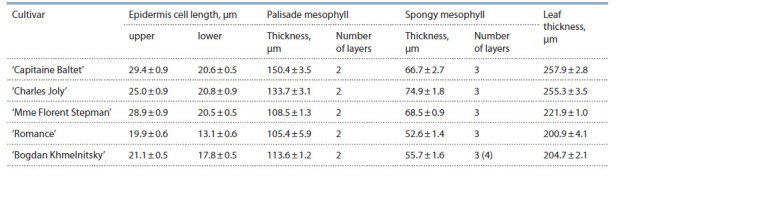
Characteristics of leaf cross-sections from the S. vulgaris cultivars

The following group of lilacs combined hybrid cultivars referred to as Hyacinthif lora, or hyacinth lilacs. These are the cultivars ‘Olimpiada Kolesnikova’, ‘Dal’nevostochnitsa’, ‘Neizvestnyi Soldat’, ‘Buffon’, ‘Esther Staley’, etc. (see Table 1). Plants from this group are distinguished both by leaf anatomical structure (Table 6) and by their resistance to P. lilacis. In Russian literature, the cultivar ‘Olimpiada Kolesnikova’ is attributed to the group of S. vulgaris cultivars (Rubtzov et al., 1980; Okuneva et al., 2008). According to the literature data, the cultivar ‘Berryer’ (S. oblata×S. vulgaris) was involved in breeding this cultivar. The mesophyll is 5-layered, consisting of 2 layers of palisade and 3(4) layers of spongy mesophylls. By its resistance to P. lilacis, the cultivar is placed in group 2 (see Table 2). The cultivar ‘Esther Staley’ bred by American lilac breeders (parents are unknown) is non-resistant to P. lilacis. The cultivar ‘Ester Staley’ was probably obtained on the basis of S. vulgaris. The structure in the cultivar ‘Neizvestnyi Soldat’ is similar to that described above, but it proved to be resistant to P. lilacis. Some cultivars of this group (‘Dal’nevostochnitsa’, ‘Tom Tayler’, and ‘Pocahontas’) are resistant to P. lilacis and have mainly 4-layered spongy mesophyll. It is likely that the resistance of the cultivars from the Hyacinthif lora group is related to the amount of genetic material obtained from parents. The 4-layered spongy mesophyll is not a trait of passive immunity, but rather serves as a marker of the presence of genetic material from S. oblata in the hybrid. 

**Table 6.**
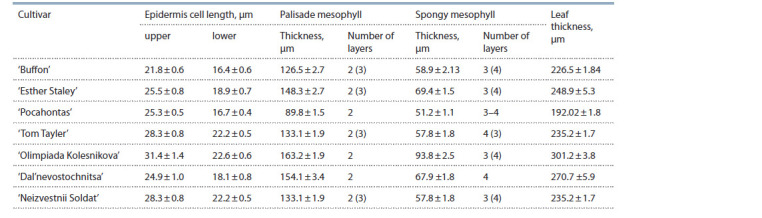
Characteristics of leaf cross-sections from lilacs of the Hyacinthif lora group

A statistical analysis (Table 7) showed the relationship between the number of spongy mesophyll layers and the degree of plants’ resistance to P. lilacis (p-value ˂ 0.001).

**Table 7.**
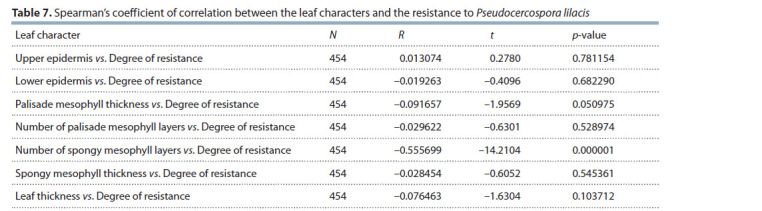
Spearman’s coeff icient of correlation between the leaf characters and the resistance to Pseudocercospora lilacis Notе. N, number of observations; R, Spearman’s correlation coefficient; t, value of the Student’s t-test for the number of degrees of freedom of n – 2; p-value, probability of error for the null hypothesis that there is no relationship between the characters.

We have found that the more layers the spongy mesophyll includes, the higher the resistance to P. lilacis (see Table 2). We have not observed any correlation relationship between the other leaf characters and the degree of resistance to P. lilacis.

The cultivars that are non-resistant to P. lilacis have the leaf anatomical structure similar to that of S. vulgaris. Thus, the number of spongy mesophyll layers can be used to predict the resistance of new lilac cultivars in the climatic conditions of southern Primorsky Krai.

According to our data, a characteristic structural feature of S. oblata and all cultivars resistant to P. lilacis is the 4-layered spongy mesophyll parenchyma. The cultivars of S. oblata subsp. oblata are similar in the ratio of heights of the first and second layers of palisade mesophyll parenchyma. The increased number of spongy mesophyll layers (4 or more) correlates (see Table 7) with the resistance to fungal disease. On the other hand, hybrids S. vulgaris×S. oblata, in which 4-layered spongy mesophyll is sometimes found, can be both resistant and non-resistant to P. lilacis. Our conclusions drawn from the examination of the leaf blade anatomical structure in lilacs are consistent with those published by Chinese breeders (Zang et al., 1983; Shuying et al., 1995). These authors reported that the inheritance of maternal traits dominates in hybrid offspring of S. oblata×S. vulgaris.

We have also found that the examination of leaf anatomy allows identification of the parents of Syringa hybrid offspring on the species level, because lilac cultivars retain the structural plan of the maternal plant. In members of the genus Cerasus Mill. (Motyleva, Dzhigadlo, 2012; Shestakova, 2013), there is a clear relationship between the primary leaf barriers and resistance to another fungal disease, coccomycosis. Contradictory data were obtained by Turovsky with co-authors (Turovsky et al., 1978), who explain the resistance of cherry tree to a fungal pathogen by the functional features of the host plant. We have established that the structural elements of the leaf anatomical structure (such as the thickness of epidermis and mesophyll) in the studied taxa of the genus Syringa are not the primary defense against infection.

## Conclusion

Certain traits of the leaf blade anatomical structure in lilacs from the subsection Euvulgaris Schneid. of the genus Syringa, such as, in particular, the spongy mesophyll parenchyma consisting of 4 layers, can be considered an indicator of the degree of their resistance to Pseudocercospora lilacis in southern Primorsky Krai. For creating lilac cultivars resistant to fungal diseases, it is expedient to cross two species (S. oblata and S. vulgaris) or their cultivars, using one of the subspecies of S. oblata as a maternal plant. With free pollination, only seeds from resistant cultivars should be taken. The difference in the mesophyll anatomical structure observed in S. oblata subsp. oblata and S. oblata subsp. dilatata can be used as an additional diagnostic trait.

## Conflict of interest

The authors declare no conflict of interest.

## References

Agroclimatic Resources of the Primorskiy Territory. Leningrad: Gidrometeoizdat
Publ., 1973. (in Russian)

Bunkina I.A., Koval E.Z., Nelen E.S. Mycoflora and Fungal Diseases
in Community Landscapes of the Far East. Vladivostok, 1971. (in
Russian)

Bykova N.B. Anatomy of the leaf and annual stem of some almond
species and hybrids. In: Biological and Structural Features of Useful
Plants in Uzbekistan (Hazelnuts, Cloves, Legumes). Tashkent: Fan
Publ., 1979;68-74. (in Russian)

Chervyakova O.N., Keldish M.A. Peculiarities of Syringa L. protection
from noxious organisms at introduction. In: Procedures of the International
Lilac сonference “International Syringa 2018”. May 21–27,
2018. Moscow; St. Petersburg, 2018;224-228. (in Russian)

Eremin G.V., Novikova L.N. Anatomical and morphological features
of hybrids between plum species with different ploidies. Doklady
VASKHNIL = Reports of the Academy of Agricultural Sciences.
1976;7:17-19. (in Russian)

Fieller E.C., Hartley H.O., Pearson E.S. Tests for rank correlation coefficients.
I. Biometrika. 1957;44(3-4):470-481. DOI 10.1093/biomet/
44.3-4.470.

Furst G.G. The structure of the seed peel in different types and varieties
of onions. Bulleten Glavnogo Botanicheskogo Sada = Bulletin of the
Main Botanical Garden. 1968;69:55-60. (in Russian)

Khomyakov M.T., Tereschenko S.I. The resistance of lilac to diseases.
Zashchita i Karantin Rasteniy = Plant Protection and Quarantine.
2000;7:31-32. (in Russian)

Mei-chen C., Lian-qing Q., Green P.S. Oleaceae. In: Flora of China.
1996;15:272-319. Available at: http://flora.huh.harvard.edu/china/
mss/volume15/index.htm

Motyleva S.M., Dzhigadlo E.N. Morphoanatomical characteristics and
elemental composition of cherry leaves in connection with resistance
to coccomycosis. Plodovodstvo i Yagovodstvo Rossii = Pomiculture
and Small Fruits Culture in Russia. 2012;30:253-261. (in Russian)

Novikova L.N. Some anatomical features of plum species and varieties.
Byulleten VIR = Bulletin of the Institute of Plant Industry ( Leningrad
). 1976;60:73-76. (in Russian)

Novikova L.N. Anatomical and morphological characteristics of the
leaf of distant hybrids of stone fruits. Nauchno-Tekhnicheskiy Byulleten
VIR = Bulletin of the Institute of Plant Industry (Leningrad ).
1982;123:54-57. (in Russian)

Okuneva I.B., Mikhailov N.I., Demidov A.S. Lilac. Collection of the
Main Botanical Garden of the Russian Academy of Sciences: History
and the Current State. Moscow: Nauka Publ., 2008. (in Russian)

Pautov A.A., Yakovleva O.V., Kolodjagnii S.F. Leafe еpidermis microrelief
in Populus (Salicaceae). Botanicheskiy Zhurnal = Botanical
Journal. 2002;87(1):63-71. (in Russian)

Pavlenkova G.A. Resistange of species of the genus Syringa L. to damaging
abiotic and biotic factors of the environment in conditions
of Orel region. In: Procedures of the International Lilac сonference
“International Syringa 2018”. May 21–27, 2018. Moscow; St. Petersburg,
2018;219-223. (in Russian)

Pham van Nang. Comparative anatomical study of some alleged hybrids
in the genus Crataegus L. Uzbekskiy Botanicheskiy Zhurnal =
Uzbek
Botanical Journal. 1976;6:48-52. (in Russian)

Plotnikova L.Ya. Plant Immunity and Breeding for Resistance to Diseases
and Pests. Moscow: KolosS Publ., 2007;69. (in Russian)

Polyakova N.V. Diseases and pests of the Syringa L. collection of the
South Ural Botanical Garden-Institute. Agrarnaya Rossiya = Agricultural
Russia. 2018;12:17-19. (in Russian)

Pshennikova L.M. Lilac Species Cultivated in the Botanical Garden-
Institute, Far Eastern Branch of the Russian Academy of Sciences.
Vladivostok: Dalnauka Publ., 2007. (in Russian)

Pshennikova L.M. Perspectives of lilac introduction in the south
of the Russian Far East. In: Procedures of the International Lilac
сonference “International Syringa 2018”. May 21–27, 2018. Moscow;
St. Petersburg, 2018;152-156. (in Russian)

Rubtzov L.I., Mikhaylov N.L., Zhogoleva V.G. Species and Varieties
of Lilac Cultivated in the USSR. Repertory Catalog. Kiev: Naukova
Dumka Publ., 1980. (in Russian)

Saakov S.G. Genus Lilac (Syringa L.). In: Trees and Shrubs of the
USSR. Moscow–Leningrad, 1960;5:435-458. (in Russian)

Shapiro S.S., Wilk M.B. An analysis of variance test for normality
(complete samples). Biometrika. 1965;52(3-4):591-611. DOI
10.1093/biomet/52.3-4.591.

Shestakova V.V. Assessment of the resistance of cherry and cherry varieties
to coccomycosis by anatomical and morphological features.
Plodovodstvo i Vinogradarstvo Yuga Rossii = Fruit Growing and
Viticulture of Southern Russia. 2010;20(2):76-82. (in Russian)

Shestakova V.V. The use of biochemical and anatomical and morphological
parameters to study the resistance of Cerasus Mill. representatives to Coccomyces hiemalis Higgins. Nauka Kubani = Science in
Kuban. 2013;1:16-20. (in Russian) 

Shkalikov V.A., Dyakov Yu.T., Smirnov A.N. et al. Plant Immunity.
Moscow: KolosS Publ., 2005. (in Russian)

Shuying Z., Yinghan F., Ronghui L. Breeding of new cultivarisin
the genus Syringa (Oleaceae). Acta Hortic. 1995;404:63-67. DOI
10.17660/actahortic.1995.404.9.

Sokolova E.A. The significance of anatomical traits for the taxonomy
of the Prunoideae (Rosaceae) subfamily. Synopsis of Dr. Biol. Sci.
Diss. St. Petersburg, 2000. (in Russian)

Tamberg T.G., Ulyanova T.N. Guidelines for Studying the Collection
of Ornamental Crops. Leningrad: Vavilov Institute of Plant Industry,
1969. (in Russian)

Tomoshevich M.A., Vorobjova I.G. Diseases of lilac in Siberian urban
plantings. Zashchita i Karantin Rasteniy = Plant Protection and
Quarantine. 2010;5:51. (in Russian)

Turovsky I.I., Zhukov O.S., Shcheglova L.A. Anatomical and ultrastructural
features of mesophyll cells of cherry forms immune and
susceptible to coccomycosis. Bulleten TsGL imeni I.V. Michurina =
Bulletin of the Michurin Central Genetic Laboratory. 1978;31:33-
36. (in Russian)

Vasilyuk V.K., Vrisch D.L., Zhuravkov A.F., Kostenko K.A., Lobanova
I.I., Mironova L.N., Petukhova I.P., Rout A.N., Seledets V.P.,
Smirnova O.A., Urusov V.M., Filatova L.D., Khmelnitsky K.A.,
Hrapko O.V., Centalovich V.T., Chipizubova M.N., Bityukov S.A.,
Pozdnyakov D.L., Voronkova N.M., Prilutsky A.N. Gardening of
Cities of the Primorskiy Territory. Vladivostok: Far Eastern Branch
of the USSR Academy of Sciences, 1987. (in Russian)

Vavilov N.I. Immunity Problems of Cultivated Plants. Vol. 4. Moscow–
Leningrad, 1964. (in Russian)

Zang S.Y., Fan Y.H., Li R.H. Hybridization and breeding of Syringa
plants. Collection of Papers on Transplanting and Domestication.
1983;3:117-121.

